# Analysis of communication and logistic processes in neonatal intensive care unit

**DOI:** 10.1186/s12887-022-03209-1

**Published:** 2022-03-15

**Authors:** J. Pirrello, G. Sorin, S. Dahan, F. Michel, L. Dany, B. Tosello

**Affiliations:** 1 Aix Marseille Univ, CNRS, EFS, ADES, Marseille, France; 2 Department of Neonatal Medicine, North Hospital, Assistance-Publique des Hôpitaux de Marseille, 13015 Marseille, France; 3 Pediatric Intensive Care Unit, Hôpital de la Timone, Assistance-Publique des Hôpitaux de Marseille, 13005 Marseille, France; 4 Aix Marseille University, LPS, Aix-en-Provence, France; 5 Service of Medical Oncology, Hôpital de la Timone, Assistance-Publique des Hôpitaux de Marseille, 13005 Marseille, France

**Keywords:** Information, Communication, Critical care, Neonatal intensive care

## Abstract

**Background:**

In neonatology, parents play a central role as guarantors of the new-born’s autonomy. Notifying parents about their infant’s status in neonatal critical care is an integral part of the care. However, conveying this information can be very difficult for physicians and the neonatal medical team.

The objective of this work is to assess the dimensions and dynamic processes of critical care communications in neonatal intensive care in order to enhance the development of theoretical and applied knowledge of these discussions.

**Methods:**

This qualitative, descriptive study was conducted on critical care new-borns less than 28 days-old who were hospitalized in a neonatal intensive care unit. Verbatim communications with the parents were recorded using a dictaphone.

**Results:**

The verbatim information had five themes: *(a)* critical care, *(b)* establishing the doctor-patient relationship, *(c)* assistance in decision making, *(d)* Socio-affective and *(e)* socio-symbolic dimensions. Our recordings underscored both the necessity of communication skills and the obligation to communicate effectively. Analysis of the dynamics of the communication process, according to the categories of delivering difficult information, showed few significant differences.

**Conclusion:**

Physician training needs to include how to effectively communicate to parents to optimize their participation and cooperation in managing their care.

## Clinical presentation

*Louis was delivered* via *caesarean section at 28 weeks in a level 3 maternity hospital because of his abnormal fetal heart rhythms and cerebral doppler bleeding in a placenta previa. Overall, his antenatal care was normal but included ultrasounds and gestational diabetes treated with insulin. A dose of corticosteroid therapy was administered just before Louis’ birth, to promote lung and brain maturation. There was no evidence suggesting any infectious etiology. Louis had difficulty adapting to extrauterine life marked by respiratory distress requiring intubation in the delivery room with installation of mechanical ventilation and a first dose of intratracheal surfactant. Note that there were signs of perinatal asphyxiation (pH 6.94 lactate 14 mmol / L). The remainder of his care was administered in the neonatal intensive care unit. His respiratory level improved rapidly and he was extubated 10 h after his birth and switched to a non-invasive, positive pressure ventilation. When Louis was admitted to the intensive care unit, the parents met with the doctors on the ward to review Louis’s situation. Two days after his birth, Louis suddenly deteriorated overnight presenting with an intra-pulmonary hemorrhage requiring reintubation. He also had signs of hemorrhagic shock which led to vascular refills and the introduction of vasopressor drugs to maintain an acceptable blood pressure. The transfontanellar ultrasound performed at the time of his degradation found a massive cerebral hemorrhage. The doctors conveyed to his parents the gravity of the situation and that a multidisciplinary collegial meeting was planned to discuss Louis’ further treatment.*

## Background

Delivering bad news to a patient requires learned skills as well as experience. The physician has a moral and legal obligation [1, 2] to communicate clinical findings to the patient, and delivering this information is integral to overall patient management. It enables developing trust, establishing a good interpersonal relationship and helps the patient and his family to appropriately participate in the decision-making process [3]. In neonatal medicine, the patient is neither able to receive and understand information nor to express his will. The infant is totally dependent on his parents as his advocates. It is the parent, that the neonatologist must inform regarding the degree of uncertainty [4, 5] and the clinical course envisioned for the new born. These elements can be considered as a whole or broken down into certain important phases or events, such as pre-admission, acute or crisis status, as well as the phases of decline or death [6]. These critical care situations include urgency, uncertainty, emotional tensions, technicalities of care, ethical dilemmas, etc. Discussing these omnipresent neonatal circumstances impact the physician/patient relationship, their decision-making capacities and enable the parent to assume their role as the patient’s advocates [7]. It becomes even more complex as physicians “have to say something they don’t want to say to someone who doesn’t want to hear it” [8]. Communicating this information in an effective manner can help engage the parents as they evaluate the ethical and health issues of their newborn’s future [9].

The objective of this work is to assess the dimensions and dynamic processes of critical care communications in neonatal intensive care in order to enhance the development of theoretical and applied knowledge in this care context.

## Method

### Design and population

This descriptive, prospective, qualitative and multicenter study was conducted in two French neonatal intensive care units, and a Pediatric Intensive Care Unit (PICU) for the management of surgical newborns. During the four-month period between November 2019 to February 2020, participating doctors recorded their communications with parents whose infants were less than 28 days old and were hospitalized in the intensive care unit. The reasons, as well as the content of the recorded communications, were left to the discretion of the doctor in order to capture the “natural” communication and to obtain a certain heterogeneity of the situation. The volunteer, participating physicians anonymously completed a form at the end of the four-month period which included their professional status, years of neonatal intensive care experience and gender.

### Selection of communication situations

The initial step was all the recorded communications that led to a modification of the child’s care, regardless of the level of “seriousness” of the situation. The other communications were called non-informative, in that they did not impact the care of the new-born.

In a second step, a retrospective selection of these recordings, which met the definition of a critical care situation, was carried out according to the following criteria: *(a)* registration of care in an “emergency” form, *(b)* extremely salient socio-symbolic issues (parenthood, “the death of a child”, disability, etc.) [10], *(c)* decisions and actions with high emotional value (emotional work by healthcare teams), *(d)* the context marked by forms of uncertainty and the progressive construction of the decision-making and information framework [11], *(e)* the confrontation of healthcare teams with important ethical and moral dilemmas [12], *(f)* the particularly “contingent” nature of the medical decision in this context.

Communication in critical care situations was categorized according to three temporalities relating to when the information was given concerning:

1. the infants’ entrance into the intensive care unit, *(2)* the diagnosis and therapeutic management, and, *(3)* the information on limiting therapeutic and comfort care upon medical refusal of unreasonable obstinacy.

### Analysis

The selected communications were transcribed and anonymized. In view of the study’s ethical declaration, only the doctors’ verbatim comments were fully transcribed. Several elements were collected for each recorded communication: the location (i.e., the family interview room, the mother’s room and the child’s room), the duration, the presence of the parent(s) (i.e., father and/or mother, or relative), the gestational age of the child (term or premature), the clinical state at the end of hospitalization (alive or deceased). Each communication was subjected to a content analysis [13], in order to identify thematic categories and formulate and classify the communications’ content based on the relevant indicators. The content analysis was done on an exploratory basis. No analysis grid was set beforehand but was built gradually during the analysis. The saturation of themes was obtained after ten communications were reviewed. The analysis and interpretation of the data were carried out individually, then collectively by a multidisciplinary group of three investigators (social psychologists and physician in the perspective of a triangulation of researchers) [14]. These analyses were carried out specifically for each recording and then transversally by its specific canvassing category. The analysis’ objective was to highlight the main themes that recur in the different categories of patients, to seek possible links and to identify communication strategies.

## Results

### Description of the population

**Fig. 1:**
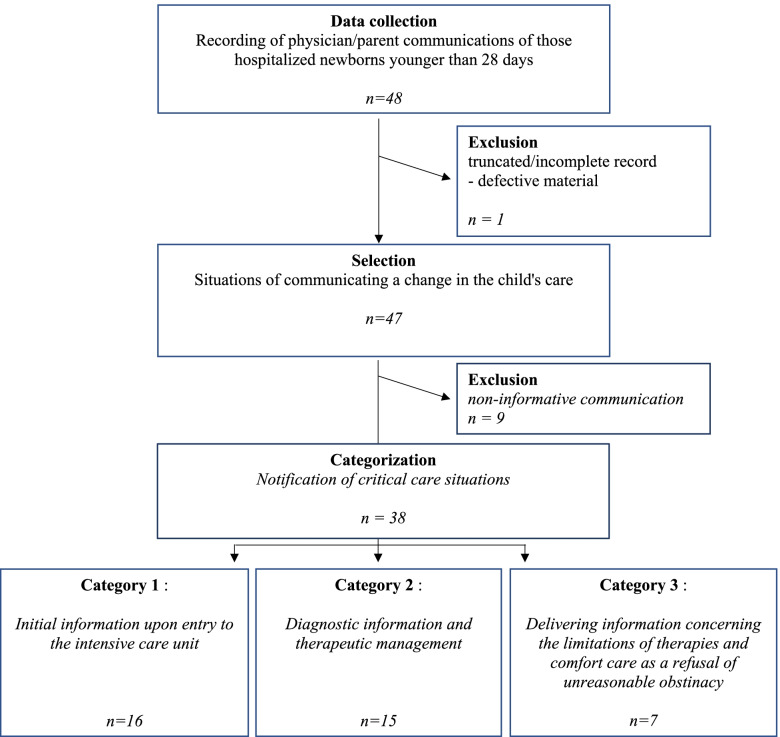
Flow diagram

A total of 48 communications were collected over the 4-month period, of which 38 corresponded to our critical care category. Within the critical care category, 16 corresponded to a first parental communication, 15 to the diagnostic information and seven to situations of therapeutic limitation (Fig. 1). The clinical settings in which these critical care outreach communications took place are summarized in Table 1. Fifteen out of 28 physicians (54%) participated in the study. The study participants were composed of 53% women and 47% male physicians. There were 69% of the participants with more than 5 years of clinical experience in neonatal intensive care unit. The recordings analysed concerned 29 new-borns with an average of 1.31 records (+/− 0.64) per new-born. Participating physicians recorded an average of 2.53 communications (+/− 2.67). The exchange between the doctor and the parent(s) either took place at the patient’s bedside (69%), in the family waiting room (29%) or in the room of the hospitalized mother (2%). The average duration of the recordings was 15.2 min. Each of our categories respectively lasted 14.6 min for first parental communication, 14.7 min for the diagnostic information and 17.9 min for situations of therapeutic limitation. The characteristics of the new-born population are grouped together in Table 2.

**Table 1:** Clinical context of announcement communications by category

Information Categories	Clinical Context	***n*** (%)
***(1) First patient status communication on admission to the intensive care unit***	Respiratory Distress	9 (56,3)
	*Transitory*	*1*
	*Hyaline membrane disease*	*4*
	*Secondary to pneumothorax*	*1*
	*Secondary to RSV bronchiolitis*	*3*
	Cerebral hemorrhage	3 (18,7)
	Anoxic-ischemic encephalopathy	1 (6,25)
	Cardiogenic shock on coarctation aorta	1 (6,25)
	Congenital malformation	2 (12,5)
	*Chylothorax*	*1*
	*Meconium cyst*	*1*
***(2) Providing details on the diagnosis and therapeutic management***	Respiratory distress	1 (6,7)
	Necrotizing enterocolitis	1 (6,7)
	Anoxo-ischemic encephalopathy	1 (6,7)
	Cerebral hemorrhage	1 (6,7)
	Neonatal convulsions	3 (20)
	Congenital malformations	5 (33,3)
	*Diaphragmatic hernia*	*1*
	*Pulmonary cyst*	*1*
	*TAPVR*^a^ *Blocked*	*1*
	*Tetralogy of Fallot*	*1*
	*Coarctation of the aorta*	*1*
	Malignant pertussis	1 (6,7)
	Supraventricular rhythm disorder	2 (13,2)
***(3) Announcement of limitations of therapies and comfort care upon medical refusal of unreasonable obstinacy***	Anoxic-ischemic encephalopathy with severe brain lesions on MRI	1 (14,3)
	Severe cerebral hemorrhage	6 (85,7)

^a^ *TAPVR* Total abnormal pulmonary venous return

**Table 2:** Characteristics of the population of newborns whose parents were informed

	***Population (n = 29)***
Premature Birth n (%)	16 (55.2)
Gestational Age (GA), average (+/− SD)	33 (+/− 6,05)
Males, n (%)	16 (55.1)
Single Pregnancy, n (%)	26 (89.6)
Length of hospital stay (days), median (IQR)^a^	13,4 (7–19)
Child’s living status at discharge, n (%)	18 (63.0)

^a^IQR *Interquartile range*

### Global thematic analysis

The thematic analysis of the full study made it possible to highlight five main themes:

1. Critical care in the clinical situation of the new-born, as well as the notion of steadfast therapeutic care and the infant’s death when clinical interventions failed.
2. The doctor(s)/parent(s) relationships were built around a common normative framework and the creation of a partnership between all participants engaged in the dialogue.
3. The decision-support theme involved the notions of uncertainty, certainty as well as the team’s thoughts.
4. The socio-emotional dimensions between the physicians and parents included empathy, emotional, reassurance and support.
5. An analysis of the sociological and symbolic dimensions that represent the child, life and pain.

Some extracts from the medical verbatim recordings appear in Table 3.

**Table 3:** Extracts of verbatim statements illustrating the different themes

Themes	Subtopics	Excerpts
***(1)*** **Critical care**	**Clinical Situation** **Unreasonable obstinacy** **Death**	*“She had a lot of trouble breathing. It took too much effort so we had to support her a lot.”* *“We know very well that it is serious, that the risk of death is very high”* *“So there, there is (*sic*) everything we do, in fact, does nothing for her. Okay? And it’s, well, it’s a medical failure, but it’s something that’s too serious for us and it doesn’t make sense to continue to do aggressive, potentially painful things.* *“There’s everything that’s gradually going towards a... a departure.”* *“It is by nature not acceptable, I, I, I know it well [...] again the death of a little baby it is not explicable, it is not admissible”*
***(2)*** **Establishing a physician-patient relationship**	**Common Framework** **Partnership**	*“We’ll keep you informed, as much as possible, of all the developments. So, it’s sometimes hard, quite raw because we have to tell you, we have to tell you the truth and* ***(****…*) *the state of your daughter at the time we see you”.* *“Do you have any questions? Any other questions? Is this clear? Do you understand?”* *“We’re all on the same page, so this is very important that we see/deal with this together.”* *“It’s up to us doctors, in alliance with you, with everything I’m doing right now, to tell you what level of care we’re going to do.*
***(3)*** **Decision making support**	**Uncertainty** **Certainty** **Team**	*“But we can’t know what will work in this child, this child (*sic*), that child”* *“We know for sure that it will result in significant sequelae, okay?”* *“I’m talking about us because it’s a team effort. The team asked me right away.*
***(4)*** **Social-emotional dimensions**	**Empathy** **Emotions** **Reassurance** **Accompagnement**	*“I understand, I understand that, I’m not in your shoes of course, but I can still understand what you’re telling me anyway”* *“You were enjoying her, the last moments of her life, with her in your arms. (Silence 25 s) (mother cries). It’s very brutal what I’m telling you, but things happen so brutally for some infants. it wasn’t really what we thought would happen, it’s not (silence 2 min) (mother cries). Do you want to go out for a while?”* *“We didn’t really expect this for _child’sname but unfortunately it happened last night.”* *“I’m really sorry, we’re all, we’re all shocked at, at what’s happening today.”* *“And you mustn’t blame yourself because you had nothing to do with it, no, but it’s important that I tell you that, for the mother, for you too, you are not responsible, you had nothing to do with it, okay?”* *“What do I know about children who have these kinds of abnormalities in the neonatal period? Well, the majority, the vast majority are fine, I can tell you that I’m not as pessimistic as I might be in other conditions, surgery, low flow.* *“The nurses are going to do some care. They’re going to take away all the medical equipment, dress her. And then you have a way to see her dressed in pajamas and not with all these machines and all these tubes. And then, uh, after that she’s going to go to the morgue.”* *“Absolutely. So, there’s that, there’s talking to her, touching her. As soon as it’s possible, you can take her against you - skin to skin.*

### Thematic analysis by category

**Fig. 2:**
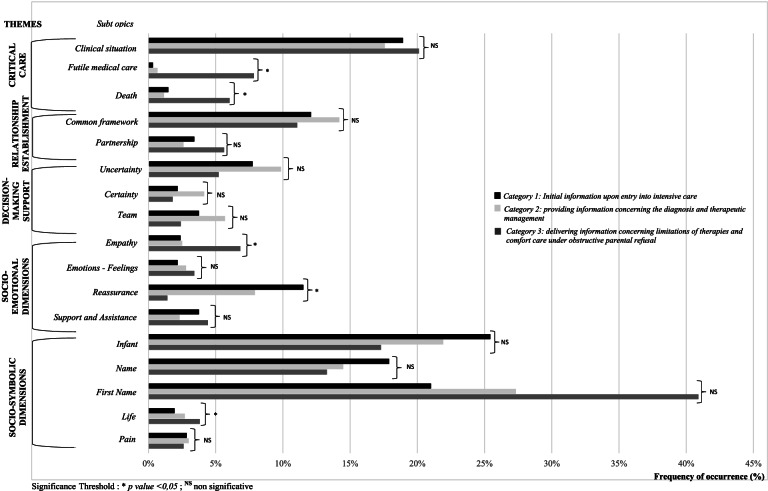
Categorization of frequency of themes by announcement category

The majority of the frequency of the subtopics was significantly different depending upon the category: life, death, therapeutic steadfastness and empathy were more significantly represented in category 3, whereas reassurance appeared in category 1 (Fig. 2). Decision support was more represented in category 2 without statistically significant differences between the three categories. The subtheme referring to the child itself was at the center of medical discussions regardless of the category. There was a more frequent (non-significant) use of the first name of the new-born within the appellation sub-topic in category 3.

## Discussion

In neonatal intensive care, there are five main themes that occur in critical care communications situations, regardless of the type and time of these discussions:

1. Critical care,
2. Establishment of the doctor-patient relationship,
3. Assistance in the decision-making process,
4. Socio-affective dimensions,
5. Socio-symbolic dimensions.

We can thus make the hypothesis of a certain standardization or even ritualization of communications in this context of care. The themes discussed, which are all corroborated within the discussions recorded by caregivers, seem to refer to what is expected in terms of communication during critical care in order to meet parental, legal and ethical expectations.

The theme centered on critical care is indisputable since it defines the field of intervention. The themes on the socio-affective and socio-symbolic dimensions refer to the communication skills required to format this information (cf. taking into account the particularity of the care situation). For example, in the most extreme critical care situations, parents are forced to confront a cruel reality, that their child’s care plan initially “contained” by uncertainty, has been replaced by a medical certainty such as death or a severe handicap that will prevent any viability in their life. Arnold et al. [15] describe those parents who receive a grim prognosis as receiving a moral distress causing them to waiver between intensive and/or comfort care, reality in the face of death and optimism despite their fears. Faced with these overwhelming realities, doctors, rather than discussing “heroic” therapies, focus their discussions around the socio-emotional aspects of the situation. The physicians’ clinical demeanour would shift to include an open expression of emotions and refocus on certain ethical values [16] including the principles of benevolence or even solicitude. Recognizing their own emotions (positive or negative), Albarracin et al. [17] show that the caregivers were able to better recognize the parents’ and families’ emotions, thus making it easier to answer the parents’ questions in a fitting way: “find the right tone in a mastered expression of one’s emotion” [18]. Categories 1 and 2 strongly depicted those issues concerning the child’s prognosis and the emotions of the parents and demonstrated that this strategy helped parents cope with their anxiety, no matter how serious the situation [3]. This strategy almost completely disappeared in category 3 for the sake of honesty, in accordance with a parental desire to receive fair and balanced information that did not convey false hopes for their child [6, 19, 20]. Thus, reassurance seems to give way to empathy and compassion, which plays a major role in establishing trust [21, 22]. Such skills must still be developed within healthcare teams in order to improve communications and reduce overall stress [23]. Finally, we were able to observe the more frequent use of the patient’s first name in the third category. This modification was perhaps involuntary, linked to a memorization bias in front of a difficult and longer hospital stay, or, on the contrary, voluntarily, with a view to humanize care in this sensitive context in order to integrally refocus care on the new-born [24]. Category 2 revealed the obligation to establish a relationship that would enable shared decision-making.

Indeed, being open-minded and proposing several options in more open discussions to make it possible to reflect and share decision-making appears to be a communication skill the parents value [25]. In order to engage in such a process, trust must be established between the doctors and the parents. Communicating about the uncertainties, regardless of the categories, can strengthen this trust. The assimilation of the information given and the management of this uncertainty over time by the parents leaves room for reflection [26]. This is fundamental in making decisions about the care plans under the best possible conditions and to keep in line with the parents’ values and beliefs as well as their child’s prognosis [27]. The evolution of a partnership between the physician and the patient is essential since, in its absence, the relationship can deteriorate [19], leading families to doubt the reliability of the information given to them [3], create conflicts and increase parental stress and anxiety. This partnership requires a willingness by both the medical professional and the family. Miller et al. have shown that maintaining good interpersonal relationships also depends on family-related factors such as the frequency of parental visits, any presence of language barriers and the level of the parents’ involvement in care [28]. With an increase in the frequency of partnering in category 3, both the caregivers and parents experience vulnerability. Discussions focus on the plans concerning their child’s life, with each side seeking to humanize their relationship. This partnership should be undertaken from the start to ensure that the keystone of shared decision-making is established [29].

A great variability in communication styles and interventions can be observed between institutions, cultures and clinicians [30]. This variability can be explained by the uniqueness of each situation, especially in the field of extreme prematurity [30]. Parents value a communication style that facilitates collaboration by offering clear and detailed information, where information is presented in a compassionate way and where there is room for questions [27, 30]. Parents also feel that a communication style based on reassurance facilitates their engagement in a trusting relationship. In this context, their interlocutor should be characterised as sensitive, compassionate, respectful and attentive to their requests and needs [19, 31]. At the same time, professionals find it difficult to adapt the types of communication according to the context and the parents. They say they favour a frame of reference based on the parents’ perceived personality, but the parents’ stress and uncertainty about the care situation can bias their judgement. To ensure that parents understand the reality of their situation, communication should be personalised taking into account sustainability, beliefs, possible disabilities and values [27, 30].

The multicentric nature of this study within the same city with its socio-economic-cultural peculiarities seems to limit the possibility of extrapolation of these data. The EURYDICE II study [32] underscored the many cultural differences concerning pediatric end-of-life issues in Europe, such as religion, culture, race, legal and professional backgrounds and other social factors. A model including several French or even European cities could have made it possible to highlight the differences in communication practices or even the emergence of new themes. Another limitation of our study is that its non-verbal aspect and the verbatim comments of the parents were not taken into account for ethical and legal reasons. This, therefore, represents a loss of information recorded on the dictaphone [12]. Nonetheless, the interest expressed in the caregivers’ words helps doctors to become aware of the importance of communication training, medical ethics and parental psychology. In a review of the literature, Berkhof et al. showed that communication training lacks standardization, has variable durations, and often combines several methods (theoretical lessons, simulation with standardized patients, group reflection a posteriori) [33].

## Conclusion

The need for communication training is recognized by medical interns, most specifically when delivering bad news [23]. In Canada, training programs for neonatal interns have already been tested. These programs emphasize the ethical dimension of situations, the perception of emotions and the values of one’s self and others [34, 35]. The inclusion of communication skills at the beginning of the medical curriculum [36] and the improvement of those skills during continuing education, by means of simulation, seem to be a major public health issue. Improving these communication skills would allow improving the physician’s interaction with patients and their families.

## Data Availability

The datasets used and /or analysed during the current study are available from the corresponding author on reasonable request.
